# Supplementary material for: Triboelectric nanogenerator sensors for soft robotics aiming at digital twin applications
Source: Nat Commun. 2020 Oct 23;11:5381. doi: 10.1038/s41467-020-19059-3 (PMC7585441; doi:10.1038/s41467-020-19059-3)
Supplement: Supplementary file 1 — Supplementary information [file 41467_2020_19059_MOESM1_ESM.pdf]

Supplementary Information for

Triboelectric Nanogenerator Sensors for Soft Robotics Aiming at Digital Twin Applications

Tao Jin, Zhongda Sun, Long Li, Quan Zhang, Minglu Zhu, Zixuan Zhang, Guangjie Yuan, Tao Chen, Yingzhong Tian*, Xuyan Hou* and Chengkuo Lee*

*Corresponding author：

Y.T. (email: troytian@shu.edu.cn), X.H. (email: houxuyan@hit.edu.cn), and C.L. (email: elelc@nus.edu.sg)

Supplementary Figures

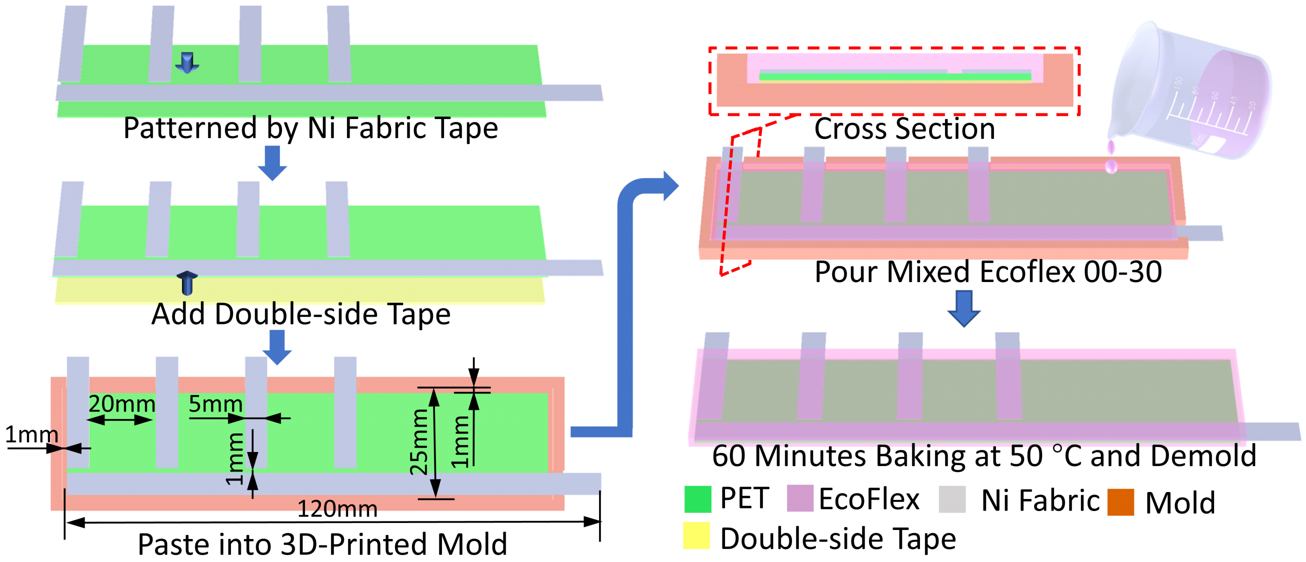

Supplementary Figure 1. The method for fabrication of the tactile TENG (T-TENG) sensor.

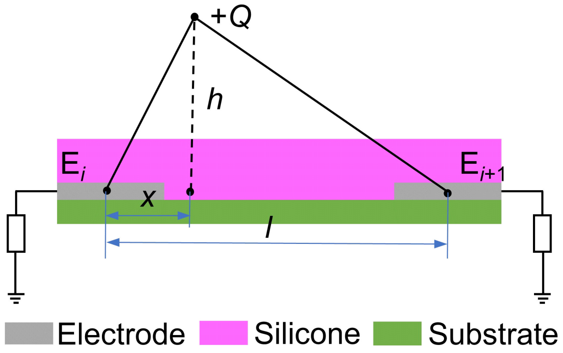

Supplementary Figure 2. The simplified diagram of the contact process for electrostatic analysis. This figure is used to express the working mechanism to detect the contact position between the adjacent electrodes of T-TENG sensor. The detailed discussion can be found in Supplementary Note 1.

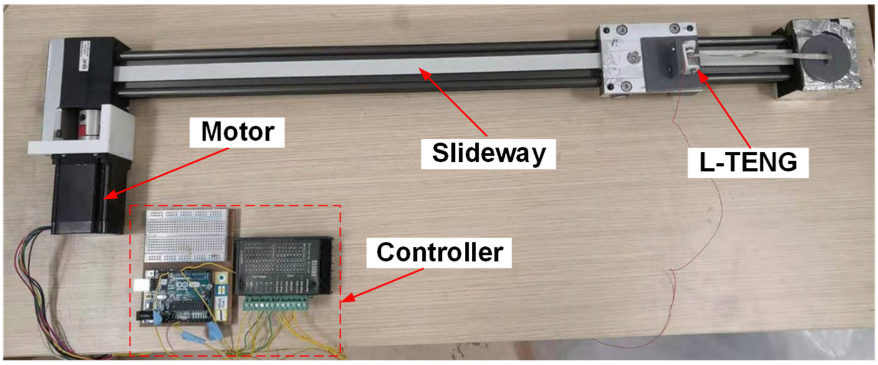

Supplementary Figure 3. Schematics of stretching and recovery machine of L-TENG sensor.

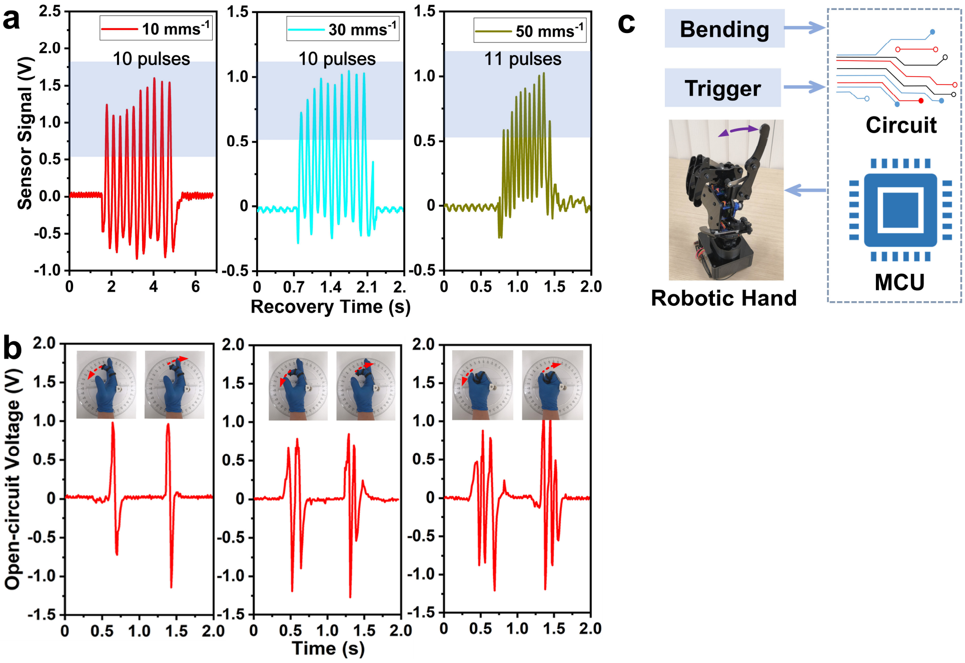

Supplementary Figure 4. The simple tests for L-TENG sensor. (a) The recovery output of the L-TENG sensor in stretching tests. **(b)** The measured output with three different bending angles (30°, 60°, 90°) when the sensor is mounted on the index finger. **(c)** Schematic diagram showing the circuit connection from the sensors to the robotic hand.

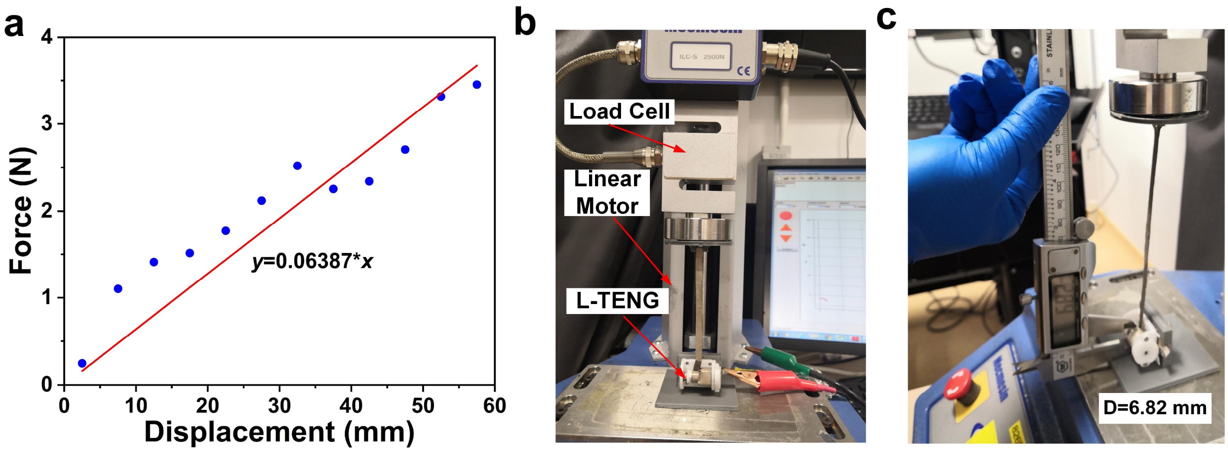

Supplementary Figure 5. The mechanics characterization of L-TENG sensor. (a) The displacement-force relationship. **(b)** The testing device. **(c)** The original diameter of L-TENG sensor.

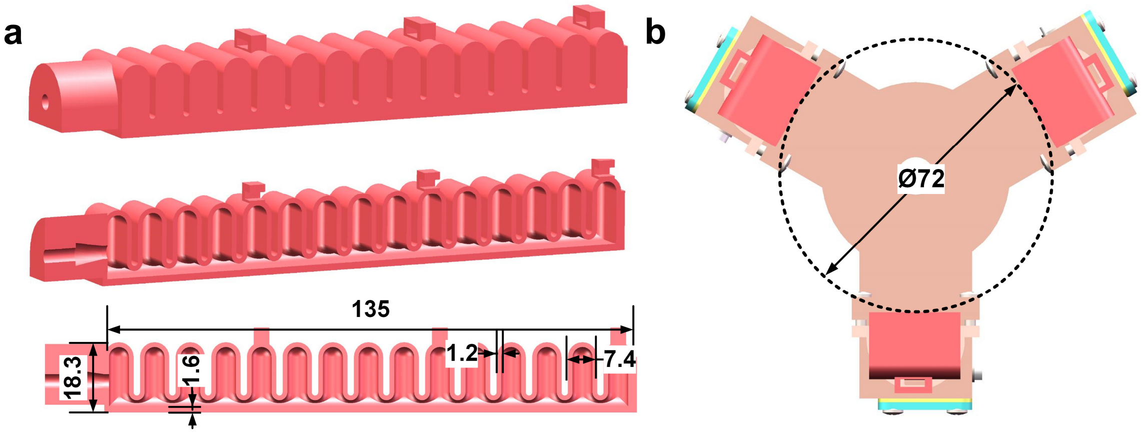

Supplementary Figure 6. The main structure of the soft gripper. (a) The parameter of singular actuator. **(b)** The layout of the soft gripper.

a

Properties	Density/g.cm^{-3}	Tensile strength/MPa	Tensile modulus/MPa	Hardness
Materials	1.19	5	12	Shore 85A

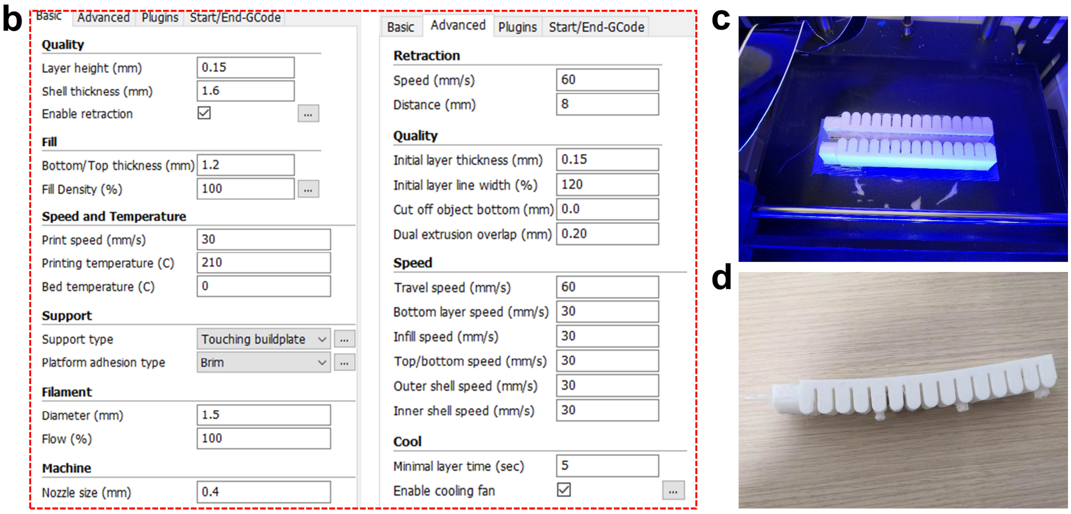

Supplementary Figure 7. The fabrication method of the soft actuator. (a) The parameter of the material. **(b)** The setting of the 3D printer. **(c)** The fabrication of the soft actuator. **(d)** The soft actuator's sample.

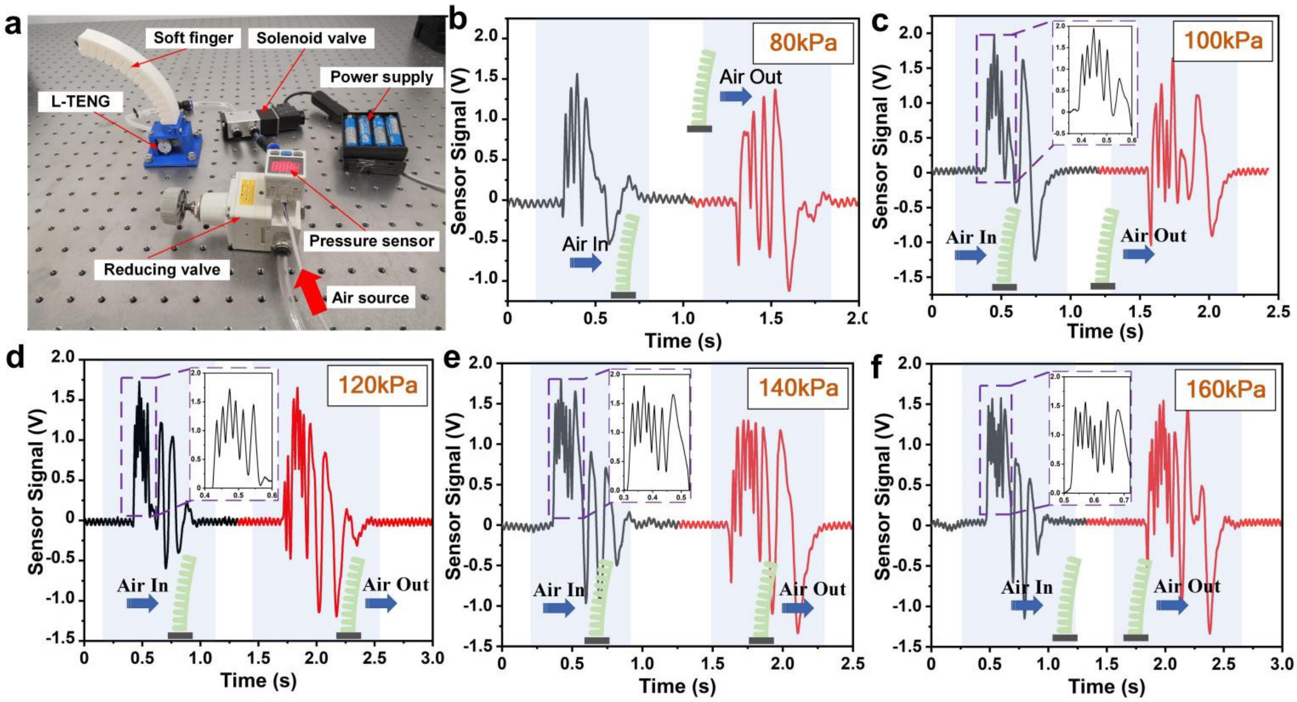

Supplementary Figure 8. The characterization of the soft actuator integrated with the L-TENG sensor. (a) The test system to control the soft actuator. **(b)-(f)** The sensor signals with different input air pressures.

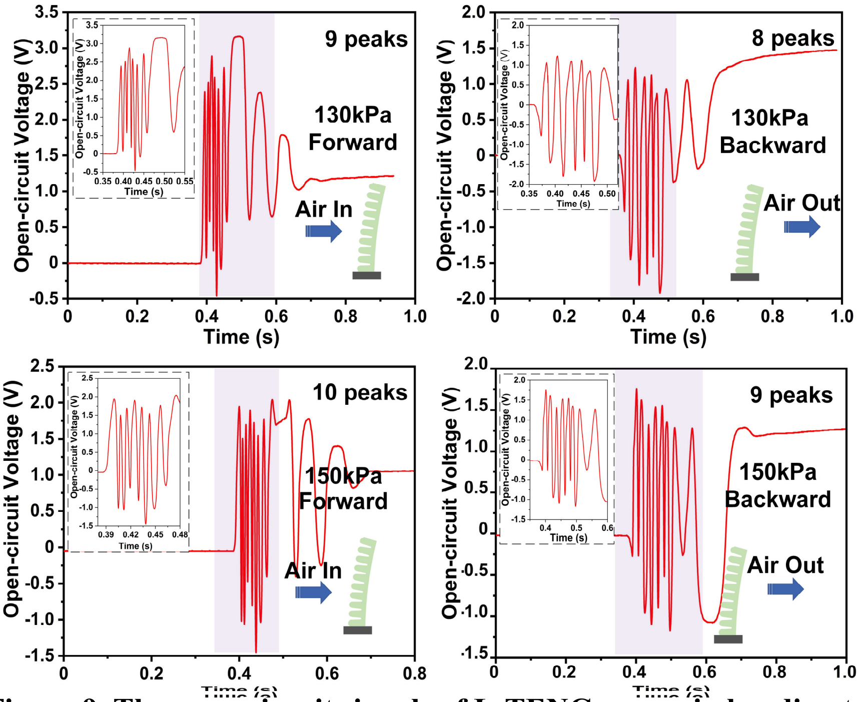

Supplementary Figure 9. The open-circuit signals of L-TENG sensor in bending tests.

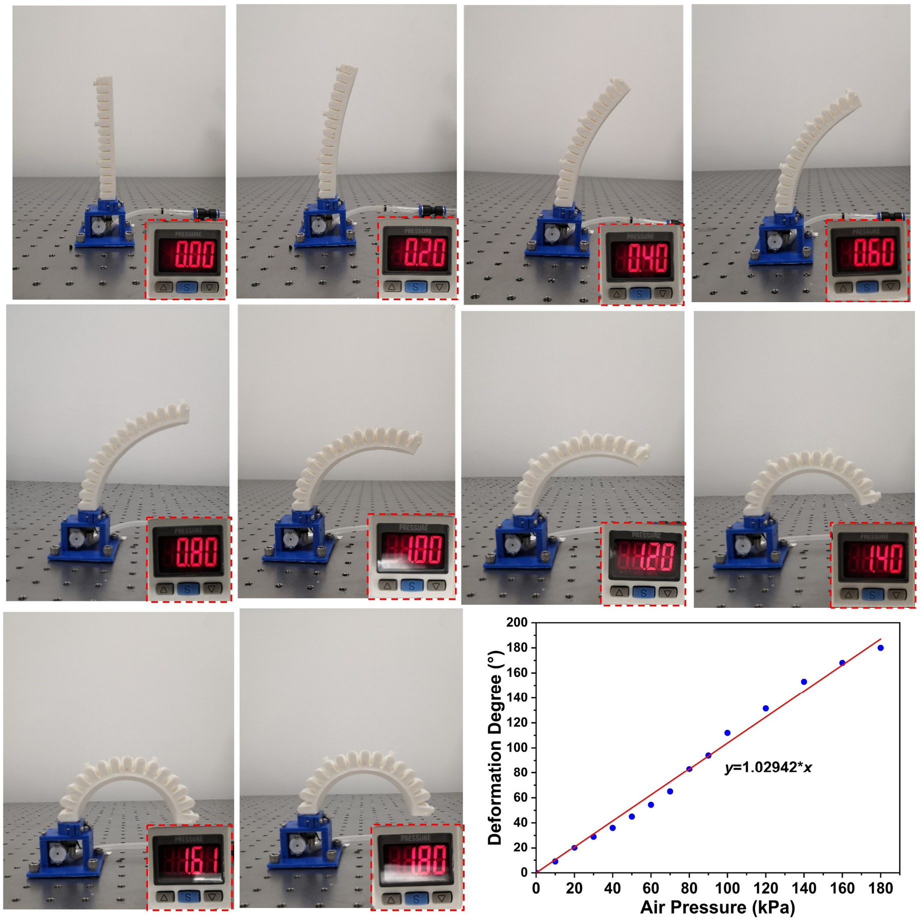

Supplementary Figure 10. Bending test of the soft actuators.

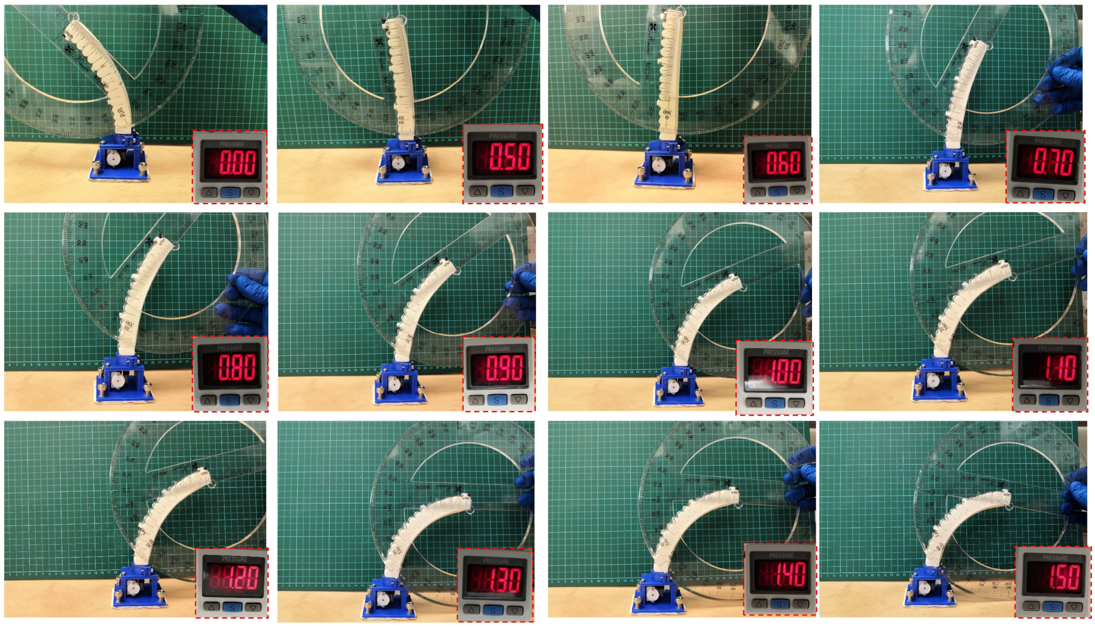

Supplementary Figure 11. Bending test of the soft actuators integrated with L-TENG sensor.

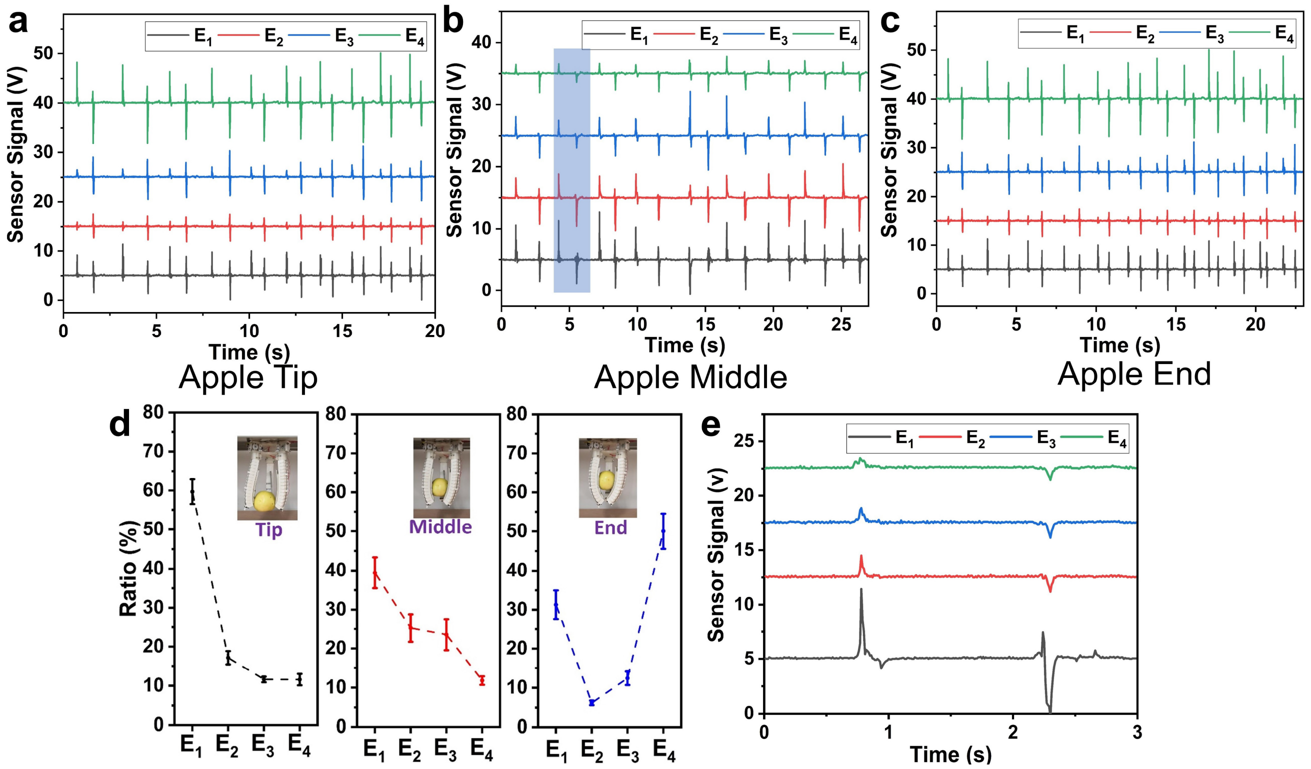

Supplementary Figure 12. Signals in gripping verification of the soft gripper integrated with T-TENG sensor. (a)-(c) contact-separation signals (9 times) when gripping and releasing the object in different contact positions. **(d)** The ratio of difference electrodes' output voltage (E_1-E_4) when gripping an apple with various contact positions. **(e)** The contact-separation signals in the shadow area of **(b)**.

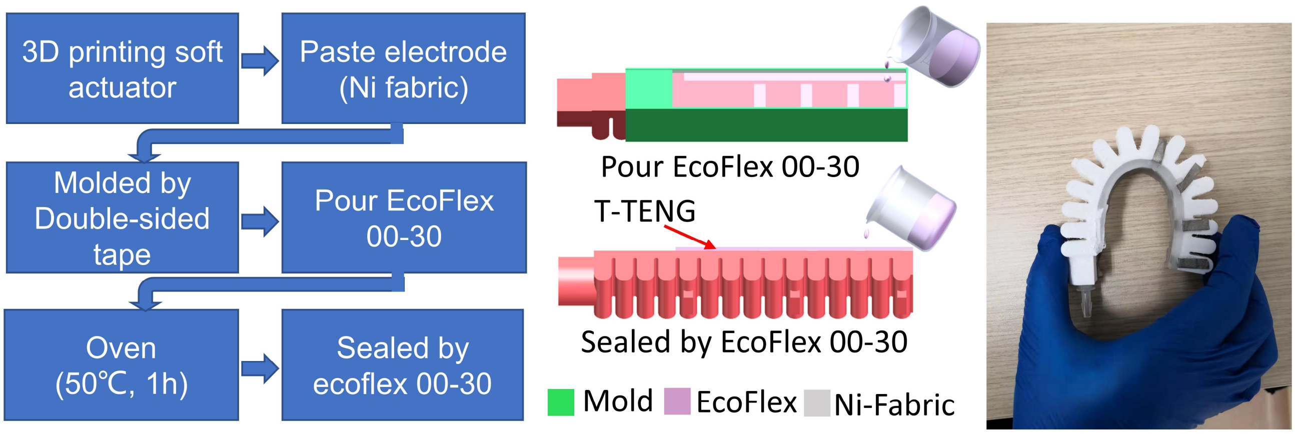

Supplementary Figure 13. The method for integrating soft actuators with T-TENG sensor.

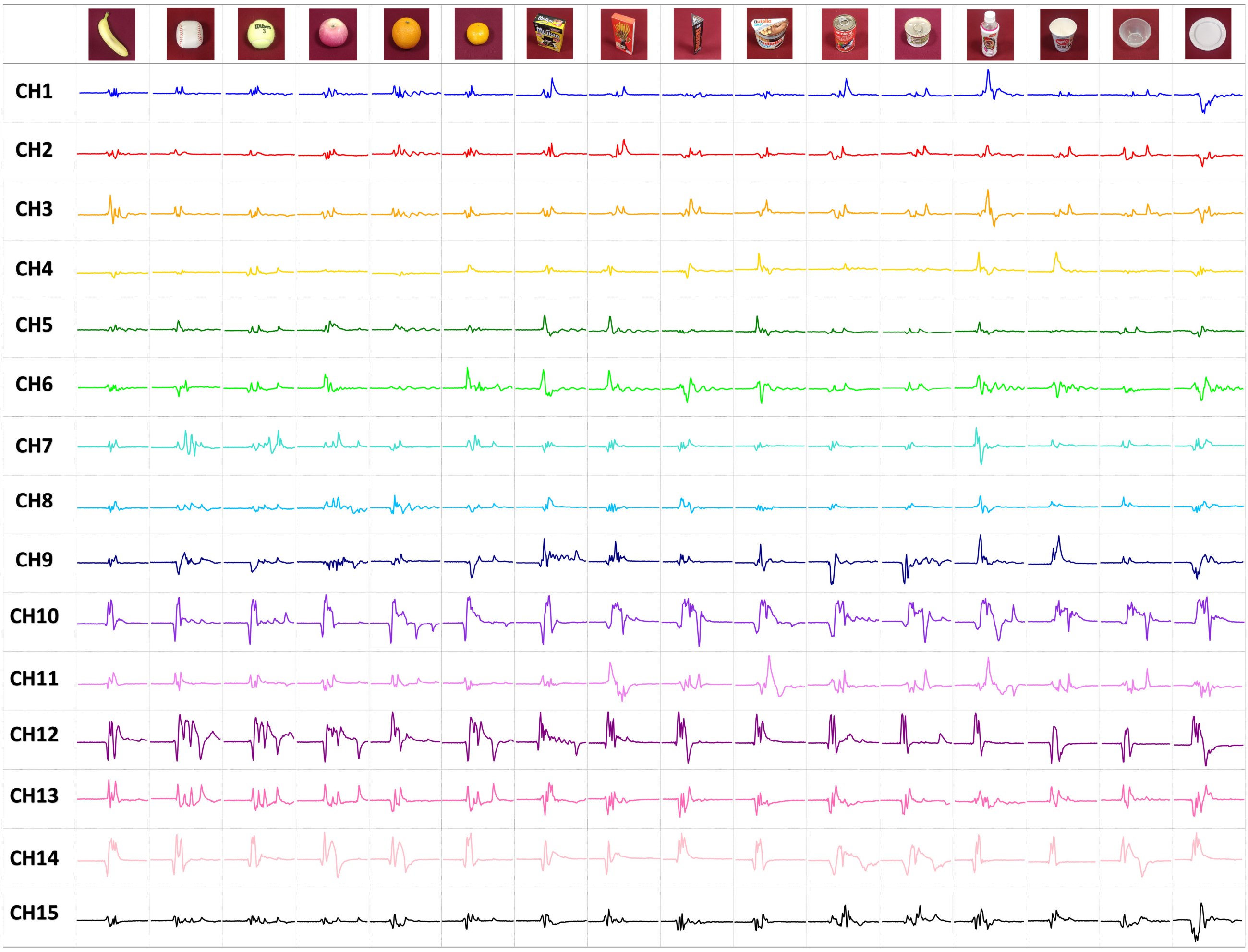

Supplementary Figure 14. The input voltage signals of 15 channels for 16 different grasped objects.

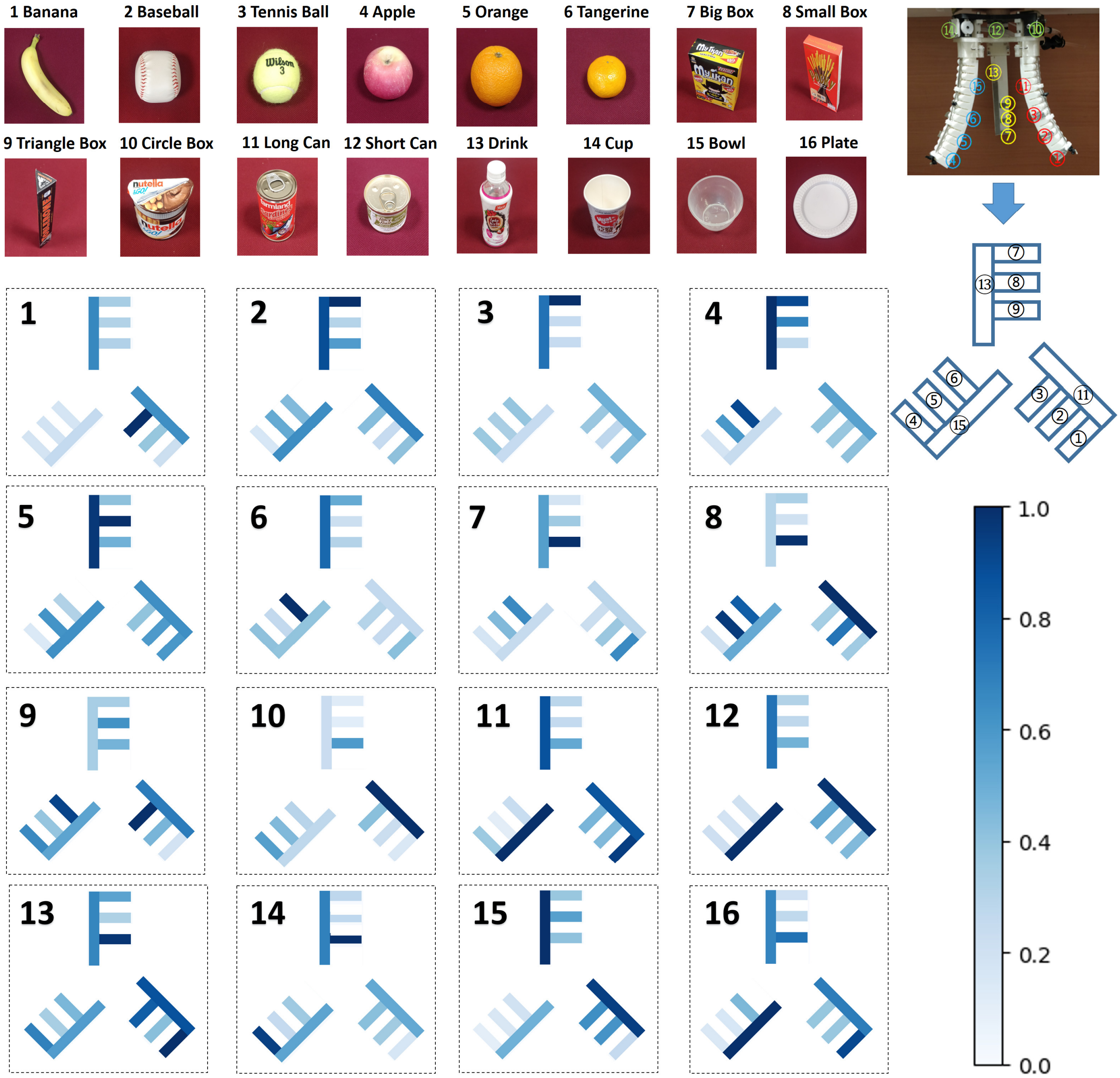

Supplementary Figure 15. Peak voltage maps of the T-TENG sensors for 16 different objects.

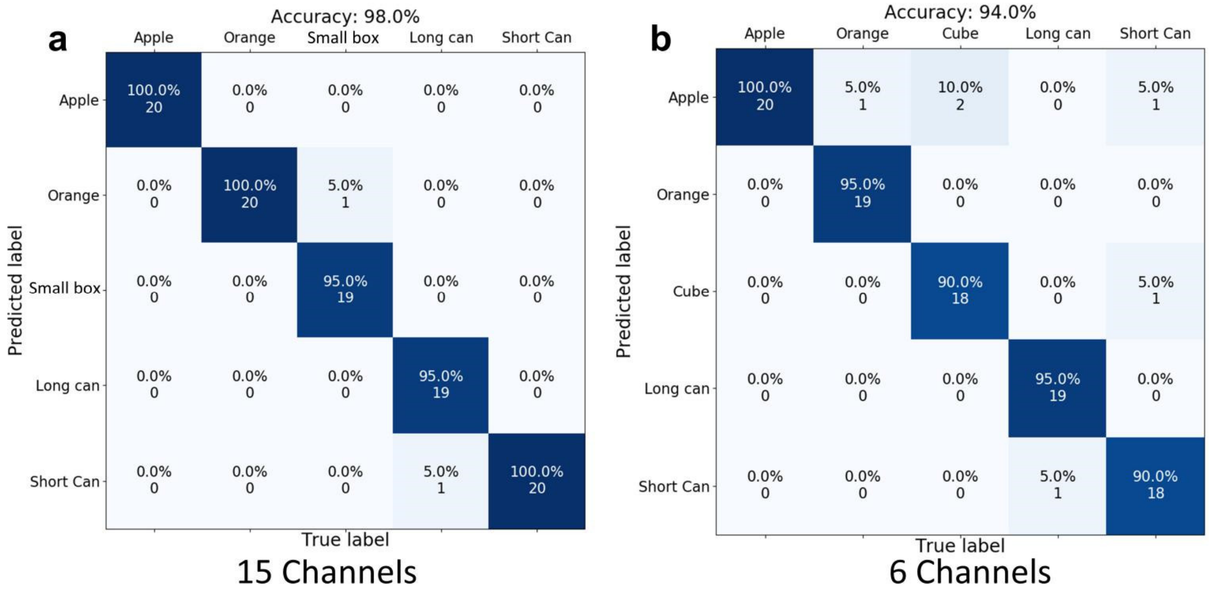

Supplementary Figure 16. The confusion maps for machine learning outcomes after trained by using different numbers of channel. (a) The confusion map after trained by using 15 channels (i.e., five channels for each soft actuator). **(b)** The confusion map after trained by using 6 channels (i.e., one T-TENG sensor (five channels) and one L-TENG sensor).

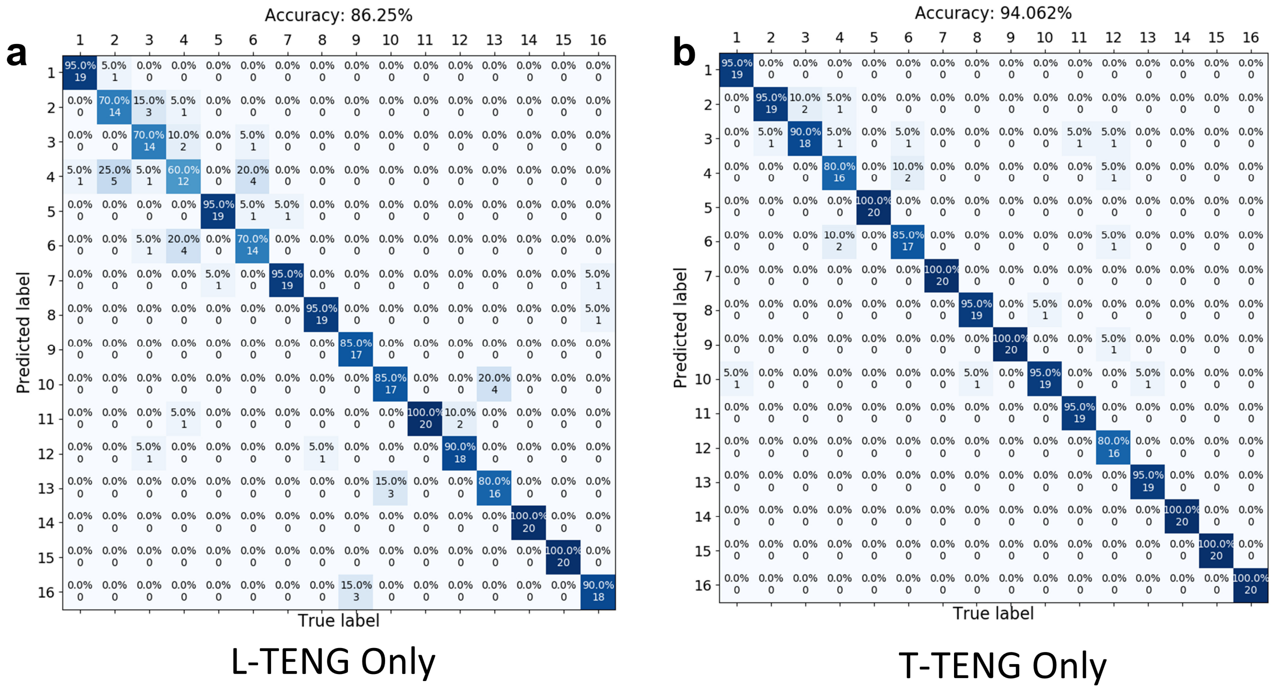

Supplementary Figure 17. The confusion maps for machine learning outcomes after trained by gripping 16 types of objects. (a) The confusion map for only using L-TENG sensors (i.e., 3 channels connected to L-TENG sensors mounted on different soft actuators). **(b)** The confusion map for only using T-TENG sensors (i.e., four channels for each soft actuator).

Supplementary Tables

Supplementary Table 1. Comparison of the tactile sensor based on various methods

Method	Position	Sliding	Contact Surface	Force	Electrode Number	Application
Triboelectric	Simple (Contact or separation)	No	No	No	One	Soft gripper[1–3]
Triboelectric	5×5 sensory array	Yes	No	No	Four	Robot control[4]
Triboelectric	8×8 sensory array	Yes	Yes	No	Sixteen	Wearable device[5]
Triboelectric	Simple (Contact or separation)	No	No	No	Two or three	Wearable device [6,7]
Capacitive, Triboelectric	4×4 sensory array	Yes	No	Normal	Eight	Humanoid Hand[8]
Piezoresistive	No	No	Yes	No	One	Soft gripper[9]
Piezoresistive	Yes	No	No	Yes	One	Soft gripper[10]
Piezoresistive	Yes	No	Yes	No	Two or more	Soft gripper[11]
Piezoelectric	No	Yes	No	No	Two	Textile detection[12]
Piezoelectric	No	No	No	Contact or not	One	Wearable device[13]
Capacitive	Yes	No	No	No	Four	HMI[14]
Capacitive	No	No	No	3-axis	Four	Humanoid Hand[15]
Capacitive	Yes	No	No	Contact or not	Six	Wearable device[16]
Thermosensitive	No	No	No	Normal	One	Humanoid Hand[17]
Optical	Yes	No	No	Normal		Sensor Mechanism[18]

Supplementary Table 2. Parameter optimization of SVM and PCA

**Classification accuracy**		**Penalty parameter C**			
		$\mathbf{1 \times 10^{-2}}$	$\mathbf{1 \times 10^{-1}}$	**1**	**10**
**Linear kernel**	PCs = 100	96.25%	95.938%	95.938%	95.938%
	PCs = 120	96.563%	96.563%	96.563%	96.563%
	PCs = 150	97.5%	97.5%	97.5%	97.5%
	PCs = 200	98.125%	97.813%	97.813%	97.813%
	PCs = 250	97.5%	97.5%	97.5%	97.5%

Supplementary Table 3. The real-time object recognition results under different temperatures

Temperature	Small box	Orange	Apple	Long Can	Short Can	Accuracy
298K	20/20	19/20	19/20	20/20	19/20	**97/100 (97%)**
313K	20/20	17/20	18/20	19/20	20/20	**95/100 (95%)**

Note: “19/20” means that 19 times correct recognition in 20 times gripping tests.

Supplementary Table 4. The test results for verifying the stability of the smart system

	Gripping Counts								
	100	300	500	700	1000	1300	1500	1800	2000
Small Box	20/20	20/20	20/20	19/20	20/20	18/20	20/20	20/20	20/20
Orange	19/20	18/20	19/20	18/20	17/20	18/20	17/20	19/20	19/20
Apple	19/20	19/20	19/20	20/20	20/20	19/20	19/20	19/20	19/20
Long Can	20/20	20/20	18/20	19/20	18/20	20/20	20/20	20/20	19/20
Short Can	18/20	20/20	20/20	20/20	19/20	20/20	19/20	19/20	19/20
**Accuracy**	**96/100 (96%)**	**97/100 (97%)**	**96/100 (96%)**	**96/100 (96%)**	**94/100 (94%)**	**95/100 (95%)**	**95/100 (95%)**	**97/100 (97%)**	**96/100 (96%)**

Note: “19/20” means that 19 times correct recognition in 20 times gripping tests.

Supplementary Notes

Supplementary Note 1. The method for sensing the contact position with improved accuracy.

It is possible for our T-TENG with improved accuracy, besides detecting the position of the middle of two electrodes or above electrodes. Here, the Supplementary Fig. 2 is harnessed to explain the contact process. According to the electrostatic induction, we can define the electric potential as

$$U = k\frac{Q}{r} \tag{1}$$

where Q is the amount of charge in the object, r is the distance to the point charge, and k is Coulomb's constant.

The distance between two nearby electrodes (E_i and E_{i+1}) is assumed to be l. So, if we assume that a charge of $+Q$ moves to the silicone rubber surface with a distance of h, the output voltage of two nearby electrodes can be expressed as

$$\begin{cases} V_i = k\dfrac{Q}{\sqrt{x^2+h^2}} - k\dfrac{Q}{x} \\ V_{i+1} = k\dfrac{Q}{\sqrt{(l-x)^2+h^2}} - k\dfrac{Q}{l-x} \end{cases} \tag{2}$$

where x represents the distance between the touch point to E_i, l-x represents the distance between the touch point and E_{i+1}, and V_i denotes the output voltages of the ith electrode.

Thus, the ratio can be derived as

$$\frac{V_{i+1}}{V_i} = \frac{\dfrac{1}{\sqrt{(l-x)^2+h^2}} - \dfrac{1}{l-x}}{\dfrac{1}{\sqrt{x^2+h^2}} - \dfrac{1}{x}} \tag{3}$$

So, if h is large enough, we can obtain

$$\frac{V_{i+1}}{V_i} = \frac{x}{l-x} \tag{4}$$

Therefore, the ratio can be used to explain the method to get a more accuracy position under various speeds and strain speeds. Similarly, as the electrode number is increased to four, we can use $\text{Ratio} = V_i / \sum_{i=1}^{4} V_i$ to sense the position.

Supplementary Note 2. The output signals for soft actuator with L-TENG sensor.

The soft actuator with L-TENG sensor was tested by the testing system as shown in Supplementary Fig. 8a. A reducing value connected with the air pressure sensor (ISE30A, SMC) was used to adjust the input air pressure, and then a two-position-three-way solenoid valve with a mechanical switch was applied to control the intake or exhaust of the soft actuator. Then, the solenoid valve was directly connected to the soft actuator or soft gripper. Here, the soft actuator integrated with L-TENG sensor was fixed on a frock and the triboelectric signal was recorded by both an ADC with concerned circuits and the electrometer (Model 6514, Keithley) connected to an oscilloscope (DSO-X3034A, Agilent). The air pressure starts from 50 kPa and increased by 10 kPa each step (50 kPa to 160 kPa) during the experiment. The result (Supplementary Fig. 8) shows some of the signals recorded by the ADC, and the whole relationship between input air pressure and the peak counting can be found in Fig. 4c. The open-circuit result in Supplementary Fig. 9 with the same peak number as the one in Supplementary Fig. 8 under the same input air pressure verifies the validity of the data. In addition, a movie (Supplementary Movie 4) shows the real-time signal tested with 130 kPa air pressure.

Supplementary Note 3. Analysis of the bending degree variations for soft actuator with L-TENG sensor or not.

It is necessary to evaluate the bending degree variations for soft actuator with L-TENG sensor or not to confirm the working space or mechanical property change caused by the L-TENG sensor. Hence, we

tested the bending capability of our 3D-printed soft pneumatic actuator to obtain the relationship between input air pressure and the corresponding bending degree of the soft actuator. The concerned control method is as same as the one in Supplementary Note 2. Note that the bending angle here is calculated by comparing to its original value (i.e., 0° for actuator without L-TENG sensor and -40° for the one with L-TENG sensor). As depicted in Supplementary Fig. 10, with the incremental air pressure, the bending degree of the soft actuator without L-TENG sensor is increasing and the relationship shows good linearity. Then, we tested the soft actuator integrated with L-TENG sensor under the same condition. As shown in Fig. 4c and Supplementary Fig. 11, although the relationship remains linearity, there is about 10° less deformation as comparing to the actuator without L-TENG sensor under the same air pressure. The reason is that the output force of the soft actuator needs to compensate the pulling force from the strip of L-TENG sensor, which can be solved by providing higher air pressure.

Supplementary Note 4. The data collecting process and the finger contact location on objects.

In terms of the primary demonstration of showing the feasibility for object recognition in warehouse or factory where the robotic gripper usually grasps products with the same position and angle, we try to maintain a relatively fixed position for 100 grasps for each object during the data collection process to achieve a better prediction result with less samples. The peak voltage maps of the T-TENG sensors for different objects shown in Supplementary Fig. 15 provide a reference for the finger contact location on the object. The peak voltages of the 12 T-TENG sensors for each sample are normalized between zero and one, and a darker color means larger contact area and contact force at this location during grasping. The grasping pressure differences among the 12 T-TENG sensors between objects are also clearly illustrated in this figure. Though the fabrication errors existing in our homemade sensors and pneumatic fingers may result in the asymmetrical distribution of the peak voltages in the T-TENG sensors for

symmetric objects, this will not affect the prediction ability of the system because both the training and testing process are done by one specific gripper and the error between different sensors is also the same for all objects. This problem can be solved in the future by using more stringent and unified fabrication standards.

Additionally, for those grasps, some of them from different objects may look similar at a certain time frame (i.e., the time of taking photo, or the time of drawing the pressure maps). However, as we mentioned in main manuscript, we are using a period of output data (200 data points at time series) of 15 channels as a training sample, which means the data includes the information of the contact force, speed, sequences, contact positions, latency, and the contact durations etc. Those multi-dimensional features, rather than the individual grasping position, will then define the identity of the grasped object. As a result, as long as those objects have distinct shape differences, the object recognition can be achieved.

Supplementary Note 5. Object recognition accuracy under changing temperature and long-term use.

Similar to other reported devices, the proposed triboelectric sensors may be influenced by changing temperature and long-term use. However, the peak counting and readout of output ration in this paper are the data processing strategies which can eliminate the absolute amplitude variations of triboelectric output caused by environmental variations, and hence, to ensure the signal stability throughout the usage. Some concerned experiments have been applied to verify this property.

Due to our application environment where the temperature seems to remain in a constant value (298K) and the fluctuation of temperature is quite small, we have compared the object recognition result under 298K and 313K. As depicted in Supplementary Table 3, the results indicate that within normal ambient temperature, our TENG sensory system is not very sensitive to the temperature. In addition, we can also

improve the accuracy of the model by getting more data under different temperatures for training to enhance the versatility of the model when environment has large temperature variation. As for recognition accuracy for long-term use, in order to reduce the proportion of necessary training and testing times in the entire stability test and make the result for certain utilization cycles more accurate, we choose to use less data: 80 samples for training for each object (total 5 objects) and 20 samples for testing for each object after cycles of utilization. As shown in Supplementary Table 4, our results indicate that though the recognition accuracy has some inevitable fluctuation due to the environmental and man-made noise, the average accuracy remains high level and there isn't an obvious decrease after gripping for 2000 times. Therefore, we believe that our design can keep a high accuracy even though after cycles of utilization and in the environment with some changing temperature.

Supplementary References

1. Chen, J., Chen, B., Han, K., Tang, W. & Wang, Z. L. A Triboelectric Nanogenerator as a Self-Powered Sensor for a Soft–Rigid Hybrid Actuator. *Adv. Mater. Technol.* **1900337**, 1–7 (2019).
2. Lai, Y. *et al.* Actively Perceiving and Responsive Soft Robots Enabled by Self-Powered, Highly Extensible, and Highly Sensitive Triboelectric Proximity- and Pressure-Sensing Skins. *Adv. Mater.* **30**, 1801114 (2018).
3. Chen, S., Pang, Y., Yuan, H., Tan, X. & Cao, C. Smart Soft Actuators and Grippers Enabled by Self-Powered Tribo-Skins. *Adv. Mater. Technol.* **1901075**, 1–10 (2020).
4. Chen, T. *et al.* Triboelectric Self-Powered Wearable Flexible Patch as 3D Motion Control Interface for Robotic Manipulator. *ACS Nano* **12**, 11561–11571 (2018).
5. Dong, K. *et al.* A Stretchable Yarn Embedded Triboelectric Nanogenerator as Electronic Skin for Biomechanical Energy Harvesting and Multifunctional Pressure Sensing. *Adv. Mater.* **30**, 1–12 (2018).
6. Yang, P. *et al.* A Flexible, Stretchable and Shape-Adaptive Approach for Versatile Energy Conversion and Self-Powered Biomedical Monitoring. *Adv. Mater.* **27**, 3817–3824 (2015).

7. Wang, H. *et al.* Self-Powered Inhomogeneous Strain Sensor Enabled Joint Motion and Three-Dimensional Muscle Sensing. *ACS Appl. Mater. Interfaces* **11**, 34251–34257 (2019).
8. Zhang, C. *et al.* A stretchable dual-mode sensor array for multifunctional robotic electronic skin. *Nano Energy* **62**, 164–170 (2019).
9. Homberg, B. S., Katzschmann, R. K., Dogar, M. R. & Rus, D. Haptic identification of objects using a modular soft robotic gripper. *IEEE Int. Conf. Intell. Robot. Syst.*, 1698–1705 (2015).
10. Thuruthel, T. G., Shih, B., Laschi, C. & Tolley, M. T. Soft robot perception using embedded soft sensors and recurrent neural networks. *Sci. Robot.* **4**, eaav1488 (2019).
11. Hughes, J. & Iida, F. Tactile Sensing Applied to the Universal Gripper Using Conductive Thermoplastic Elastomer. *SOFT Robot.* **5**, 512–526 (2018).
12. Liao, Z. *et al.* A tactile sensor translating texture and sliding motion information into electrical pulses. *Nanoscale* **7**, 10801–10806 (2015).
13. Deng, W. *et al.* Cowpea-structured PVDF/ZnO nanofibers based flexible self-powered piezoelectric bending motion sensor towards remote control of gestures. *Nano Energy* **55**, 516–525 (2019).
14. Peele, B. *et al.* Untethered Stretchable Displays for Tactile Interaction. *SOFT Robot.* **6**, 142–149 (2019).
15. Liang, G., Wang, Y., Mei, D., Xi, K. & Chen, Z. Flexible Capacitive Tactile Sensor Array with Truncated Pyramids as Dielectric Layer for Three-Axis Force Measurement. *J. Microelectromechanical Syst.* **24**, 1510–1519 (2015).
16. Ai, Y. *et al.* All rGO-on-PVDF-nanofibers based self-powered electronic skins. *Nano Energy* **35**, 121–127 (2017).
17. Li, G. & Zhu, R. A Multisensory Tactile System for Robotic Hands to Recognize Objects. *Adv. Mater. Technol.* **1900602**, (2019).
18. Massari, L. *et al.* A Machine-Learning-Based Approach to Solve Both Contact Location and Force in Soft Material Tactile Sensors. *SOFT Robot.* **00**, 1–12 (2019).
