# Supplementary material for: Triboelectric nanogenerator sensors for soft robotics aiming at digital twin applications
Source: Nat Commun. 2020 Oct 23;11:5381. doi: 10.1038/s41467-020-19059-3 (PMC7585441; doi:10.1038/s41467-020-19059-3)
Supplement: Supplementary file 3 — Description of Additional Supplementary Files [file 41467_2020_19059_MOESM3_ESM.pdf]

Description of Additional Supplementary Files

File name: Supplementary Movie 1
Description: Open-circuit stretching test for L-TENG sensor

File name: Supplementary Movie 2
Description: Open-circuit bending test when the L-TENG sensor is mounted on the index finger

File name: Supplementary Movie 3
Description: TENG-based glove used to control robotic hand

File name: Supplementary Movie 4
Description: Open-circuit bending test for the soft actuator integrated with L-TENG sensor

File name: Supplementary Movie 5
Description: Real-time gripped object prediction based on real-time signals and trained model

File name: Supplementary Movie 6
Description: Object recognition using machine learning technology and real-time projection in the digital twin.
